# Prevalence and Severity of Sleep Disorders in Patients With Mycosis Fungoides: A Case‐Control Study

**DOI:** 10.1155/jskc/5528328

**Published:** 2025-12-28

**Authors:** Ahmad Vafaeian, Aidin Shahilooy, Maryam Daneshpazhooh, Robabeh Abedini, Farid Mohamadi, Ala Ehsani, Hamidreza Mahmoudi

**Affiliations:** ^1^ Autoimmune Bullous Diseases Research Center, Razi Hospital, Tehran University of Medical Sciences, Tehran, Iran, tums.ac.ir; ^2^ School of Medicine, Shahid Beheshti University of Medical Sciences, Tehran, Iran, sbmu.ac.ir

**Keywords:** ISI, mycosis fungoides, PSQI, sleep quality

## Abstract

**Introduction:**

Mycosis fungoides (MF) is the most common form of cutaneous T cell lymphoma. Given that MF is a rare disease, evidence regarding its impact on sleep and quality of life is limited; however, our study aims to evaluate this aspect.

**Patients and Methods:**

In this case‐control study, 72 patients with MF were enrolled in the case group, and 72 matched healthy individuals were enrolled in the control group. Data regarding sleep disturbances were collected and analyzed using the Pittsburgh Sleep Quality Index (PSQI) and Insomnia Severity Index (ISI) questionnaires. Poor sleep quality was defined as a score > 5.

**Results:**

The control and case groups had median scores of 5.12 ± 2.57 and 10.22 ± 3.75 by PSQI and 6.39 ± 4.86 and 16.28 ± 6.99 by ISI, respectively. Multiple logistic regression revealed a significant association between the study group and the prevalence of poor sleep quality, as measured by both the PSQI (*p* < 0.0001) and the ISI (*p* < 0.0001). However, age, sex, marital status, pruritus, disease stage and onset, and lesion locations were not found to be associated with poor sleep quality.

**Discussions:**

Both the severity and prevalence of sleep disorders were significantly higher in the patients compared to a matched healthy population. Due to the profound impact of sleep on quality of life and considering the high prevalence of sleep disorders in patients with MF, evaluating a patient’s sleep quality could improve their quality of life. Therefore, professional treatment can be administered if sleep disorders are observed or suspected.

## 1. Introduction

Mycosis fungoides (MF) is the most common form of cutaneous T cell lymphoma (CTCL), accounting for approximately one‐half of all CTCL cases [1]. Although MF most commonly affects the skin, lymph nodes, blood, and visceral organs may also be involved.

Disease etiology is yet to be discovered. Recent studies have proposed genetic predisposition and epigenetic factors [2, 3]. Skin lesions in the classic form of the disease include patches or plaques that may be localized or widespread, tumors, and erythroderma, which are usually seen in sun‐protected parts of the patient’s body. Clinical and histopathological studies are usually used to make a diagnosis, but when the findings are inconsistent, immunohistochemistry and molecular data are required to confirm the diagnosis [4–6]. MF is the most prevalent in individuals over the age of 50 [7–9]. The incidence of the disease is higher in men than in women [7–10].

Skin problems and sleep problems are closely related. The skin plays an important role in sleep physiology by regulating body temperature and initiating sleep [11]. Meanwhile, skin‐related symptoms such as itching and mental health issues may also interfere with sleep [12]. According to the recent studies, CTCL patients suffer from insomnia, sleep disorders, and daytime sleepiness [13–15].

Various subjective sleep evaluation methods are available; however, only a few can be used to assess sleep quality and disorders in patients with cancer [16–18]. The most commonly used sleep disorder measurement instruments for the general population are the Insomnia Severity Index (ISI), Epworth Sleepiness Scale, and Pittsburgh Sleep Quality Index (PSQI) [19].

The PSQI is a reliable evaluation questionnaire with internal validity that has been used in both the general population and populations with different clinical diagnoses. In addition, this questionnaire has been used to evaluate sleep quality in cancer patients and their relatives [18, 20–22]. Multiple studies have demonstrated the compatibility and validity of this questionnaire with various populations, including the elderly, the normal population, and cancer patients [19, 20, 23, 24].

The ISI questionnaire is another tool to assess insomnia in patients. It consists of 7 questions with a 5‐point Likert scale and scores the severity and effects of insomnia in the last 2–4 weeks. The questionnaire was used in the general population and in patients with cancer, and it showed high sensitivity and specificity [25–27]. Different studies have recommended scores of 10 [28], 8, and 11 [29, 30] as the threshold for screening insomnia. Different populations have examined and confirmed the validity of this questionnaire, which indicates its reliability for assessing sleep problems in various clinical studies [27].

Given that MF is a rare disease, evidence regarding its impact on sleep and quality of life is limited; however, our study aims to evaluate this aspect. Using the PSQI and ISI questionnaires, this study evaluated the prevalence and severity of insomnia and sleep‐related symptoms in patients with MF. We have also evaluated their association with patient demographics and clinical characteristics and compared these data with those of the general population based on the PSQI and ISI.

## 2. Methods

### 2.1. Study Population

The study was conducted at Razi Tertiary Skin Hospital, Tehran University of Medical Sciences, Tehran, Iran, from January 2021 to December 2021. This study adhered to the Declaration of Helsinki and received approval from the Tehran University of Medical Sciences Ethics Review Board. All patients autonomously signed an informed consent form free from coercion.

This observational case‐control study included 72 patients with definite clinical and histological diagnoses of MF, who were referred to the hospital’s outpatient or phototherapy clinic. As a control group, 72 individuals who were matched to the patients regarding age, gender, marital status, occupation, and place of residence were also recruited. This study did not include patients with a prior history of insomnia, sleep disorders, mental disorders, or psychiatric medication use before the MF diagnosis. Healthy individuals suffering from acute or chronic skin conditions, chronic systemic illnesses, or psychiatric disorders were not recruited either. Sociodemographic and clinical information was gathered for each patient from their admission records.

### 2.2. Questionnaires

During an interview with each participant, a physician filled out the PSQI and ISI questionnaires in order to evaluate the sleep quality.

PSQI consists of 19 questions evaluating seven domains: subjective sleep quality (1 question), sleep latency (2 questions), sleep duration (1 question), habitual sleep efficiency (3 questions), sleep disturbances (9 questions), use of sleep medication (1 question), and daytime dysfunction (2 questions). Each question has a response scale with scores ranging from 0 to 3, where 0 means “very good,” 1 means “fairly good,” 2 means “fairly bad,” and three means “very bad.” Based on the above components, a subjective sleep quality score between 0 and 21 is calculated. Higher scores indicate poor sleep quality and high levels of sleep disorders. A global score of five or eight or more indicates significantly poor sleep quality in both the general population and patients with cancer [31–33]. A score of 10 may be used to measure clinical insomnia [34].

The ISI questionnaire comprises seven questions rated on a 5‐point Likert scale (0 = not at all, 4 = very severe) and aims to assess the severity and effects of insomnia over the past 2–4 weeks. This questionnaire evaluates seven domains of sleep: 1: difficulty falling asleep (examining the early stages of sleep), 2: difficulty staying asleep (middle stages of sleep), 3: waking up early (late stages of sleep), 4: satisfaction with sleep status, 5: interference with daily functioning, 6: significance of sleep problems by others, and 7: patient anxiety about the severity of sleep problems. In accordance with the above components, a subjective sleep quality score between 0 and 28 is obtained. Poor sleep quality, based on the questionnaire results, was defined as a score greater than 5.

### 2.3. Other Data Gathering

Additional data, including age, sex, marital status, presence of pruritus, time to disease onset, and lesion location, were collected. Disease staging was based on the extent of skin, lymph node, and visceral organ involvement, as this has been shown to correlate with prognosis and overall life expectancy [35, 36].

### 2.4. Study Endpoints

The primary endpoint was the comparison of sleep quality between patients with MF and the control group, as measured by PSQI and ISI scores. Secondary endpoints included associations between sleep quality and demographic or disease characteristics (e.g., stage, lesion location, and pruritus), as well as the correlation between PSQI and ISI scores.

### 2.5. Statistical Analysis

Mean ± SD was used to present results for quantitative variables with a normal distribution, medians ± IQR for variables not following a normal distribution, and N (%) for qualitative variables. The Shapiro–Wilk test was used to assess the normality of variables.

Multivariate logistic regression was used to compare the prevalence of poor sleep quality between the study groups and other variables of interest. The analysis was also performed in the case group to additionally assess the effects of lesion locations and disease stage. Furthermore, Wilcoxon rank–sum tests were used to assess the effect of the study groups on each PSQI component. Kendall’s Tau was used to assess the correlation between ISI and PSQI scores. All the analyses were performed in the R open‐source environment (R Foundation for Statistical Computing, Vienna, Austria).

## 3. Results

### 3.1. Demographic and Clinical Characteristics

The study included 144 participants, evenly divided between the case and control groups. The mean age of the total population was 45.03 ± 18.47 years, with 45.25 ± 18.39 years for the control group and 45.25 ± 18.39 years for the case group. In the study, 71 individuals (49.31%) were female. In the control group, 36 individuals (50.00%) were female, and in the case group, 35 (48.61%) individuals were female. Most participants were married (73.61%). The median disease duration was 24.00 ± 39.00 years (Table 1).

**Table 1:** Demographic and clinical characteristics of the study population based on sleep quality assessed by PSQI and ISI scores.

Variable			Total	Sleep quality based on PSQI > 5		Sleep quality based on ISI > 5	
				Normal	Poor	Normal	Poor
Total	Age, years	Mean ± SD	45.03 ± 18.47	43.35 ± 18.06	45.89 ± 18.71	43.10 ± 18.08	45.82 ± 18.65
	Sex	Female	71 (49.31%)	24 (48.98%)	47 (49.47%)	17 (40.48%)	54 (52.94%)
		Male	73 (50.69%)	25 (51.02%)	48 (50.53%)	25 (59.52%)	48 (47.06%)
	Marital	Single	38 (26.39%)	15 (30.61%)	23 (24.21%)	14 (33.33%)	24 (23.53%)
		Married	106 (73.61%)	34 (69.39%)	72 (75.79%)	28 (66.67%)	78 (76.47%)
	Pruritus	−	136 (94.44%)	48 (97.96%)	88 (92.63%)	41 (97.62%)	95 (93.14%)
		+	8 (5.56%)	1 (2.04%)	7 (7.37%)	1 (2.38%)	7 (6.86%)
	Group	Control	72 (50.00%)	42 (85.71%)	30 (31.58%)	34 (80.95%)	38 (37.25%)
		Case	72 (50.00%)	7 (14.29%)	65 (68.42%)	8 (19.05%)	64 (62.75%)

Case	Age, years		45.25 ± 18.39	47.00 ± 13.17	45.06 ± 18.94	41.25 ± 13.25	45.75 ± 18.96
	Sex	Female	35 (48.61%)	3 (42.86%)	32 (49.23%)	3 (37.50%)	32 (50.00%)
		Male	37 (51.39%)	4 (57.14%)	33 (50.77%)	5 (62.50%)	32 (50.00%)
	Marital	Single	18 (25.00%)	2 (28.57%)	16 (24.62%)	3 (37.50%)	15 (23.44%)
		Married	54 (75.00%)	5 (71.43%)	49 (75.38%)	5 (62.50%)	49 (76.56%)
	Pruritus	−	64 (88.89%)	6 (85.71%)	58 (89.23%)	7 (87.50%)	57 (89.06%)
		+	8 (11.11%)	1 (14.29%)	7 (10.77%)	1 (12.50%)	7 (10.94%)
	Disease onset, years	Median ± IQR	24.00 ± 39.00	12.00 ± 39.50	24.00 ± 48.00	12.00 ± 38.25	24.00 ± 48.00
	Stage	1A	50 (69.44%)	5 (71.43%)	45 (69.23%)	7 (87.50%)	43 (67.19%)
		1B	19 (26.39%)	2 (28.57%)	17 (26.15%)	1 (12.50%)	18 (28.12%)
		2B	3 (4.17%)	0 (0.00%)	3 (4.62%)	0 (0.00%)	3 (4.69%)
	Lower ext.	−	22 (30.56%)	2 (28.57%)	20 (30.77%)	2 (25.00%)	20 (31.25%)
		+	50 (69.44%)	5 (71.43%)	45 (69.23%)	6 (75.00%)	44 (68.75%)
	Upper ext.	−	46 (63.89%)	5 (71.43%)	41 (63.08%)	6 (75.00%)	40 (62.50%)
		+	26 (36.11%)	2 (28.57%)	24 (36.92%)	2 (25.00%)	24 (37.50%)
	Trunk	−	27 (37.50%)	1 (14.29%)	26 (40.00%)	3 (37.50%)	24 (37.50%)
		+	45 (62.50%)	6 (85.71%)	39 (60.00%)	5 (62.50%)	40 (62.50%)
	Head and neck	−	60 (83.33%)	4 (57.14%)	56 (86.15%)	7 (87.50%)	53 (82.81%)
		+	12 (16.67%)	3 (42.86%)	9 (13.85%)	1 (12.50%)	11 (17.19%)

For the case group, 11.11% of participants experienced pruritus. The lesion locations were as follows: lower extremities in 69.44% of participants, upper extremities in 36.11%, trunk in 62.50%, and head and neck in 16.67% (Table 1).

### 3.2. Sleep Quality Assessment

The PSQI scores had means of 7.67 ± 4.10 for the total population, 5.12 ± 3.25 for the control group, and 10.22 ± 6.25 for the case group. Similarly, the ISI scores were 11.33 ± 7.78 for the total population, 6.39 ± 8.00 for the control group, and 16.28 ± 9.25 for the case group.

Based on the PSQI scores, 95 (65.97%) participants experienced poor sleep quality, with 30 (31.58%) in the control group and 65 (68.42%) in the case group. Similarly, using ISI scores, 102 participants (70.83%) reported poor sleep quality, with a higher prevalence among cases (64, 62.75%) versus controls (38, 37.25%) (Table 1). Furthermore, the PSQI and ISI scores were positively correlated (Tau: 0.769, *p* < 0.0001).

### 3.3. Association of Variables With Poor Sleep Quality

For PSQI‐defined poor sleep quality, being in the case group was significantly associated with higher odds of poor sleep quality (OR: 2.62, 95% CI: 1.72–3.68, *p* < 0.0001). Other variables were not significantly associated with PSQI‐defined poor sleep quality, including age (*p* = 0.646), sex (*p* = 0.812), marital status (*p* = 0.895), and presence of pruritus (*p* = 0.740) (Table 2).

**Table 2:** Logistic regression analysis of the impact of features on clinical poor sleep quality.

	Feature	Feature level	PSQI > 5			ISI > 5		
			Estimate	95% CI	*p*	Estimate	95% CI	*p*
Total	Age		0.01	−0.03, 0.04	0.646	0.00	−0.03, 0.04	0.898
	Sex	Female	0.10	−0.72, 0.92	0.812	0.66	−0.15, 1.49	0.113
	Marital	Married	0.09	−1.32, 1.51	0.895	0.47	−0.90, 1.87	0.501
	Pruritus	+	−0.38	−2.37, 2.63	0.740	−0.16	−2.14, 2.86	0.890
	Group	Case	2.62	1.72, 3.68	< 0.0001^∗∗∗∗^	2.04	1.17, 3.05	< 0.0001^∗∗∗∗^

Case	Age		−0.03	−0.11, 0.04	0.428	0.02	−0.05, 0.08	0.645
	Sex	Female	0.60	−1.32, 2.81	0.547	0.63	−0.99, 2.38	0.452
	Marital	Married	1.22	−1.90, 4.67	0.453	0.56	−2.09, 3.39	0.680
	Pruritus	+	0.00	−0.02, 0.03	0.910	0.01	−0.01, 0.05	0.368
	Disease onset		−0.29	−3.21, 3.19	0.849	−0.84	−3.73, 2.60	0.575
	Stage	1A						
		1B	0.08	−2.10, 2.55	0.942	1.25	−1.09, 4.55	0.352
		2B	15.66	−119.37, NA	0.994	14.33	−144.76, NA	0.995
	Lower ext.	+	−0.54	−3.02, 1.51	0.627	−0.83	−3.11, 1.00	0.412
	Upper ext.	+	0.89	−1.40, 3.67	0.474	0.37	−1.69, 2.85	0.740
	Trunk	+	−1.34	−4.52, 0.97	0.296	−0.11	−1.99, 1.72	0.906
	Head and neck	+	−1.86	−4.12, 0.14	0.072	0.05	−2.19, 3.14	0.970

Abbreviations: CI, confidence interval; ISI, Insomnia Severity Index; PSQI, Pittsburgh Sleep Quality Index.

^∗∗∗∗^ *p* < 0.0001.

^∗∗∗^ *p* < 0.001.

^∗∗^ *p* < 0.01.

^∗^ *p* < 0.05.

Similarly, for ISI‐defined poor sleep quality, case status was significantly associated with increased odds (OR: 2.04, 95% CI: 1.17–3.05, *p* < 0.0001). Similarly, other variables did not show significant associations, including age (*p* = 0.898), sex (*p* = 0.113), marital status (*p* = 0.501), and the presence of pruritus (*p* = 0.890) (Table 2).

Among cases, disease onset, disease stage, and affected body areas were not significantly associated with poor sleep quality by either measure. For PSQI‐defined poor sleep quality: disease onset (*p* = 0.849), stage (*p* = 0.942 for 1B, *p* = 0.994 for 2B), lower extremities (*p* = 0.627), upper extremities (*p* = 0.474), trunk (*p* = 0.296), and head and neck (*p* = 0.072). For ISI‐defined poor sleep quality: disease onset (*p* = 0.575), stage (*p* = 0.352 for 1B, *p* = 0.995 for 2B), lower extremities (*p* = 0.412), upper extremities (*p* = 0.740), trunk (*p* = 0.906), and head and neck (*p* = 0.970) (Table 2).

### 3.4. PSQI Subdomain Analysis

A comparison of PSQI subdomain scores between the case and control groups revealed the areas most affected by the disease. Subjective sleep quality was worse in the case group (median 2.00) compared to the control group (median 1.00) (*p* < 0.0001). Insomnia was more severe in the case group (median 2.00) versus the control group (median 1.00) (*p* < 0.0001). Sleep disturbances were more frequent in the case group (median 2.00) than in the control group (median 1.00) (*p* < 0.0001). Daytime dysfunction was more severe in the case group (median 3.00) compared to the control group (median 1.00) (*p* < 0.0001).The use of sleep medication was higher in the case group than in the control group (*p* = 0.030). No significant differences were observed between groups in sleep duration (*p* = 0.252) or sleep efficiency (*p* = 0.128) components (Table 3).

**Table 3:** Comparative analysis of PSQI score components between study groups.

PSQI components	Total median + IQR	Control median + IQR	Case median + IQR	Estimate^§^	95% CI	*p*
Subjective sleep quality	1.50 ± 1.00	1.00 ± 1.00	2.00 ± 1.00	−1.00	−2.00, −1.00	< 0.0001^∗∗∗∗^
Insomnia	1.00 ± 1.00	1.00 ± 1.00	2.00 ± 1.25	−1.00	−1.00, −1.00	< 0.0001^∗∗∗∗^
Sleep duration	0.00 ± 1.00	0.00 ± 1.00	0.00 ± 1.00	0.00	0.00, 0.00	0.252
Sleep efficiency	0.00 ± 0.00	0.00 ± 0.00	0.00 ± 0.00	0.00	0.00, 0.00	0.128
Sleep disturbances	1.00 ± 1.00	1.00 ± 0.00	2.00 ± 1.00	−1.00	−1.00, −1.00	< 0.0001^∗∗∗∗^
Sleep medication	0.00 ± 1.00	0.00 ± 0.00	0.00 ± 1.00	0.00	0.00, 0.00	0.030^∗^
Daytime dysfunctions	2.00 ± 2.00	1.00 ± 1.00	3.00 ± 1.00	−1.00	−2.00, −1.00	< 0.0001^∗∗∗∗^

Abbreviations: CI, confidence interval; PSQI, Pittsburgh Sleep Quality Index.

^§^Difference of the location parameters.

^∗∗∗∗^ *p* < 0.0001.

^∗∗∗^ *p* < 0.001.

^∗∗^ *p* < 0.01.

^∗^ *p* < 0.05.

## 4. Discussion

MF is a rare disease, and the presentation of a series of 72 cases with histological diagnoses and clinical staging may significantly contribute to the current understanding of this condition. In this case‐control study, we evaluated and compared the prevalence and severity of sleep disorders in patients with MF to those in the healthy group using two validated questionnaires. We found that patients with MF experience sleep problems more frequently and with greater severity. For the PSQI subdomains, subjective sleep quality, insomnia, sleep disturbances, daytime dysfunction, and the use of sleep medication were significantly impacted by MF.

In this study, we observed an increased frequency of poor sleep quality according to the PSQI and ISI questionnaires, with adjusted odds ratios of 2.62 and 2.04, respectively. None of the questionnaires revealed any association between the prevalence of poor sleep quality and age, gender, presence of pruritus, or marital status in the general population. For patients with MF, no effect was observed regarding disease stage, disease onset, or lesion locations. Similar findings were reported in a systematic review conducted in 2015 on studies that used PSQI as a data collection tool [24].

Sleep disorders and problems negatively impact patients’ quality of life and have been linked to psychological problems such as depression and increased suicide risk [37, 38]. It has been shown that patients with MF experience higher levels of depression and anxiety compared to the control group [39, 40]. Sleep disorders are also one of the most common complaints of cancer patients [41]. Nonmelanoma skin neoplasms are associated with sleep disorders. Insomnia (22.8%) and breathing problems (18.7%) have been observed in patients with nonmelanoma skin cancers [41].

MF is the most common CTCL that requires lifelong management. In a study of 930 CTCL patients, 89% of whom had MF, 59.9% complained of sleep problems after diagnosis, and 21.3% described their sleep disorder as severe [42]. Sixty‐five point seven percent of patients reported experiencing fatigue during waking hours, with 23.8% describing it as severe [42]. Another study of CTCL patients published in 2020 found that 61.1% had sleep disorders and 66% had fatigue during waking hours [43].

Regarding the effect of disease duration on sleep quality, there are various results reported depending on the underlying disease. While in patients with ankylosing spondylitis [44], chronic kidney disease [45], and Type II diabetes [46], longer disease duration was correlated with worse sleep quality; however, for rheumatoid arthritis and Parkinson’s disease, this was not the case [47, 48]. We did not find any significant effect of increased disease duration of MF on the prevalence of poor sleep quality.

In our study, we did not find any association between higher disease stages and an increased prevalence of poor sleep quality. In addition, we did not observe any correlation between the location of the lesions and sleep quality. Although more advanced disease stages have been reported to be associated with diminished quality of life, evidence of the exact effect of the MF stage on sleep quality is lacking [49]. Furthermore, the involvement of certain locations, such as the head and neck, has been reported to be associated with a worse prognosis [50]. This may be due to the effects of treatment in later stages, psychological adaptations, and a lack of correlation between disease stage and sleep‐disrupting signs and symptoms.

Some studies present findings that contrast with ours [51]. One possible reason for this discrepancy could be the heterogeneity of the populations studied, as certain correlations only emerged in stratified settings. For example, it has been shown that age and PSQI scores are correlated only in women aged 50–79 or in individuals with specific occupations [51, 52]. Our study was neither designed nor sufficiently powered to conduct such stratified analyses. Furthermore, differences in the underlying diseases among different study populations may account for the variations in reported scores, as studies have shown that populations with different types of underlying conditions, particularly various cancers, show varying PSQI scores [24]. Similar factors may explain the lack of effect from pruritus on the prevalence of poor sleep in our study despite its common association with sleep disturbances in the literature [53, 54]. Also, other factors not included in this study, such as mental health status, could confound the effects of pruritus and disease stage on sleep quality [55].

In this study, we observed similar outcomes when using the ISI and PSQI. Previous research has demonstrated that these two tools have comparable reliability and diagnostic validity [56]. However, the PSQI, with its greater number of questions and longer completion time, is less effective for quickly assessing the severity of insomnia [56]. In the domains of sleep disorders measured by the PSQI, we observed that the case group had significantly higher severity in five out of seven areas, indicating the extent of sleep disorders and highlighting the specific areas most affected in patients with MF.

The main limitation of our study was the low‐to‐moderate size of both the patient and control groups, primarily due to the low prevalence of MF in the community. If we had waited to employ common strategies for matched controls, such as R‐to‐one matching [57], it would have significantly prolonged the study’s enrollment process. For the disease stage, which had three categories, this issue was more pronounced, reducing the power to analyze the correlation between disease stages and sleep quality. In addition, our patient population was not vastly racially and geographically diverse, and the questionnaires were only completed at a single point in time. Additional studies with a longitudinal design to understand sleep quality changes over time, particularly in association with the progression of the disease and application of various treatments, are mandatory. Also, we used PSQI and ISI forms for evaluation, while disease‐specific instruments incorporating cutaneous lymphoma factors may better capture sleep disorders in these patients. Furthermore, we did not stratify the population based on the treatment received, which could have confounded the results. Specifically for pruritus, only 8 participants (5%) in our study population experienced it, demonstrating both the effect of treatment and also limiting the power to analyze the correlation between pruritus and sleep quality. Furthermore, pruritus, as a categorical variable, could have limited the accuracy of the results, and further studies using refined continuous metrics are needed to improve accuracy. In addition, other factors that may affect sleep, such as mental health status, should be considered in future studies to provide a more comprehensive understanding of sleep disturbances in this population.

## 5. Conclusions

It was shown that sleep disorders have a high prevalence among patients with MF and are one of the most common complaints of these patients. The prevalence and severity of sleep disorders, according to both questionnaires, were significantly higher in these patients than in the normal population. Slightly less than half of MF patients had clinical insomnia, which indicates the importance of paying attention to the sleep problems when treating these patients and helping to reduce the severity of sleep disorders in these patients, which can have positive effects on their quality of life.

## Ethics Statement

The study was approved by the Ethics Committee of Tehran University of Medical Sciences with the approval ID: IR.TUMS.MEDICINE.REC.1397.850 (https://ethics.research.ac.ir/ProposalCertificateEn.php?id=49716).

## Disclosure

All authors have read and approved the final manuscript.

## Conflicts of Interest

The authors declare no conflicts of interest.

## Author Contributions

All authors contributed to the final editing of the manuscript text. All authors contributed to conceptualization. Ahmad Vafaeian, Aidin Shahilooy, and Hamidreza Mahmoudi performed the research. Ahmad Vafaeian and Aidin Shahilooy wrote the manuscript draft. Ahmad Vafaeian created the tables and performed data cleaning and statistical analyses. Maryam Daneshpazhooh, Robabeh Abedini, and Hamidreza Mahmoudi supervised the study.

Ahmad Vafaeian and Aidin Shahilooy contributed equally to the work and should be considered co‐first authors.

## Funding

This research did not receive any specific grant from funding agencies in the public, commercial, or not‐for‐profit sectors.

## Data Availability

The data that support the findings of this study are available from the corresponding author upon reasonable request.
